# Real-Time Crystal Growth Monitoring of Boric Acid from Sodium or Lithium Sulfate Containing Aqueous Solutions by Atomic Force Microscopy

**DOI:** 10.1021/acsomega.2c06953

**Published:** 2023-03-20

**Authors:** Wilson Alavia, Andreas Seidel-Morgenstern, Dana Hermsdorf, Heike Lorenz, Teófilo A. Graber

**Affiliations:** † Faculty of Engineering, Universidad Alberto Hurtado, Almirante Barroso 10, 8340575 Santiago, Chile; ‡ Max Planck Institute for Dynamics of Complex Technical Systems Magdeburg, Sandtorstraße 1, D-39106 Magdeburg, Germany; § Institute for Process Engineering, Otto von Guericke University Magdeburg, Universitätsplatz 2, D-39106 Magdeburg, Germany; ∥ Departamento de Ingeniería Química y Procesos de Minerales, Universidad de Antofagasta, 1270300 Antofagasta, Chile

## Abstract

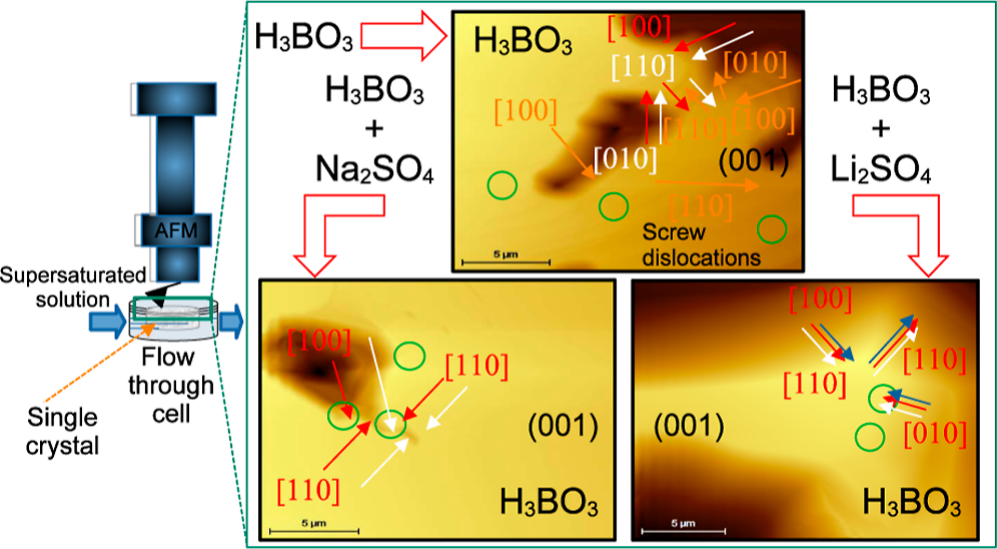

The crystal growth of boric acid from an aqueous solution in the absence and presence of sodium and lithium sulfate was studied by real-time monitoring. For this purpose, atomic force microscopy in situ has been used. The results show that the growth mechanism of boric acid from its pure and impure solutions is spiral growth driven by screw dislocation and that the velocity of advancement of steps on the crystal surface, and the relative growth rate (ratio of the growth rate in presence and absence of a salt) is reduced in the presence of salts. The reduction of the relative growth rate could be explained by the inhibition of advancement of steps of the (001) face mainly in the growth direction [100] caused by the adsorption of salts on the actives sites and the inhibition of the formation of sources of steps such as dislocations. The adsorption of the salts on the crystal surface is anisotropic and independent of the supersaturation and preferentially on the active sites of the (100) edge. Moreover, this information is of significance for the improvement of the quality of boric acid recovered from brines and minerals and the synthesis of nanostructures and microstructures of boron-based materials.

## 1 Introduction

Boric acid is recovered from boron ores (borax, colemanite, and ulexite)^1^ and brines of salt lakes that contain boron species.^2,3^ The main boron reserves are located in Turkey, Russia, Chile, China, and USA.^4^ Colemanite (CaO·2B_2_O_3_·6H_2_O) is used in Europe^1^ and ulexite (Na_2_O·2CaO·5B_2_O_3_·16H_2_O) in Chile^5^ and Argentina.^6^ In Chile, boric acid is produced from ulexite of the Salar de Surire^5^ and brines from the Salar de Atacama.^2,3^ The brines contain high concentrations of K^+^, Na^+^, Mg^2+^, Ca^2+^, B^3+^, Li^+^, SO_4_^2–^, and Cl^–^ ions.^7^

The process to recover boric acid from brines is composed of sequential steps of concentration (by solar evaporation), purification, crystallization, drying, and packing. However, this process has drawbacks. At the crystallization step, the presence of ionic species remaining in the feed solutions up to this step affects the boric acid quality.^8^ For example, the presence of sodium sulfate influences the yield and decreases the growth rate of boric acid; therefore, the mean size of crystals decreases,^9^ and the presence of lithium sulfate decreases the purity of boric acid because this salt co-precipitates as Li_2_SO_4_·H_2_O.^2^

The impurity effect is also present when recovering boric acid from boron ores. For example, when borax is reacted with sulfuric acid to form boric acid in solution, sodium sulfate is produced. This salt co-precipitates with boric acid during cooling crystallization, also affecting the boric acid purity.^10^ Besides the technical issues promoted by the impurities (chlorides, sulfates, iron, calcium, and arsenic), their presence decreases the product price and, thus, has an impact on its market.^6^ Therefore, it is necessary to deal with the impurity effect to produce high-quality boric acid crystals.

To address the impurity effects on the boric acid crystallization, the solubility, metastable zone width, and crystal growth dynamics in the presence of the relevant species have to be investigated. Besides, the knowledge of the surface phenomena occurring during the crystal growth, in the absence and presence of impurities, is useful to validate theoretical models^11,12^ for the optimization of crystallization processes to obtain a desired shape, purity, and crystal size distribution.

In the literature, solubility, metastable zone width, and growth rate data for aqueous solutions saturated in boric acid in the presence of sodium sulfate^9^ and sodium chloride^13^ have been reported. In a previous work, we reported the solubility,^14,100^ density, viscosity, electrical conductivity, and refractive index of solutions saturated in boric acid of the systems H_3_BO_3_ + Na_2_SO_4_ + H_2_O^15^ and H_3_BO_3_ + Li_2_SO_4_ + H_2_O^14^ at *T* = 293.15, 298.15, 303,15, 308.15, and 313.15 K. It was found that the salts and temperature affect the boric acid solubility, increasing in the presence of sodium sulfate and decreasing in the presence of lithium sulfate at a constant temperature. The salt effect on these properties is significant, increasing with salt concentration increments at a constant temperature.

Also, we presented a theoretical explanation of the reduction of the growth rate of boric acid in the presence of sodium sulfate;^16^ this salt was found to behave as very mobile impurity, which adsorbed on active sites of the crystal surface moderately; therefore, the growth rate is not inhibited completely. However, experimental work that addresses this problem is necessary. The crystal growth rate of boric acid may be measured in the presence of the salt and other impurities. Further, the growth mechanism of boric acid in the presence of impurities and their adsorption on the growth surface of boric acid during the crystal growth should be determined.

The crystal growth of boric acid has been studied by different methods. Batch,^9^ fluidized bed,^17,18^ and rotating disc^19^ techniques have been used to evaluate the growth rate. Those methods can estimate the overall growth rate, but they are not appropriate to determine the mechanisms involved in the growth of specific crystal faces. The growth rate and growth mechanisms of specific crystal faces have been studied using a temperature-controlled flow cell placed under an optical microscope,^20−23^ but it is limited to image growth processes at the scale equivalent to the crystal faces.^22^

Since atomic force microscopy (AFM) provides the ability to image crystal growth and dissolution processes in situ at micrometer- and nanometer-scale resolutions,^24−26^ it has been widely used to investigate growth mechanisms^27,28^ and mechanisms of kink formation,^29^ the transition between growth mechanisms,^30^ and the effects of additives, pollutants, and impurities.^24,31−36^ Further, it has been applied to study the role of impurities in surface modification,^34^ to distinguish between polymorphs for pharmaceutical compounds,^37^ to depict the kinetics of crystallization of amorphous solids by targeting a crystal at the surface,^38^ to study the crystallization and adsorption of compounds on oxide surfaces^26^ and the crystallization behavior of films at different temperatures,^39^ and to determine the relative stability of crystal faces.^40,41^

Therefore, this work presents the real-time monitoring of the crystal growth of boric acid from boric acid aqueous solutions in the absence and presence of Na_2_SO_4_ or Li_2_SO_4_ by AFM in situ, with the aim to determine the effect of these impurities on the boric acid growth mechanism and relative growth rate. The AFM apparatus enables continuous crystallization at a constant temperature. To identify suitable boric acid growth faces and their growth directions in the preparation of the AFM measurements, single crystal growth cell studies were performed under an optical microscope.

This work is organized as follows. Section 2 presents the methods used to study the crystal growth of boric acid. Section 3 analyzes the crystal growth cell and AFM in situ results and presents the main findings. Finally, Section 4 states the conclusions.

## 2 Methods

### 2.1 Materials

All solutions were prepared by mass, using an analytical balance with an uncertainty of ±1 × 10^–4^ g (Denver Instrument Co. Model AA-200). The reagents used were H_3_BO_3_ (Acros Organics, 99.99%), Na_2_SO_4_ (Sigma-Aldrich, ≥99.0%), and Li_2_SO_4_·H_2_O (Sigma-Aldrich, 99.0% dry basis). Boric acid, sodium sulfate, and lithium sulfate were dried to constant weight in an oven at 60, 100, and 120 °C, respectively. Distilled and deionized water produced with a Milli-Q Plus apparatus (Millipore Bedford, MA, USA) was used in all procedures.

### 2.2 Preparation of Single Crystals of H_3_BO_3_ for Single-Crystal Growth Cell and AFM Growth Experiments

Single crystals of boric acid were grown by the evaporation of an aqueous solution saturated in boric acid saturated at 20 °C. The solutions were evaporated at room temperature inside a desiccator for 20 days. The resultant crystals were flat hexagonal without internal fractures and around 2 mm in size (see Figure 4b).

### 2.3 Single-Crystal Growth Cell Studies

**Figure 1:**
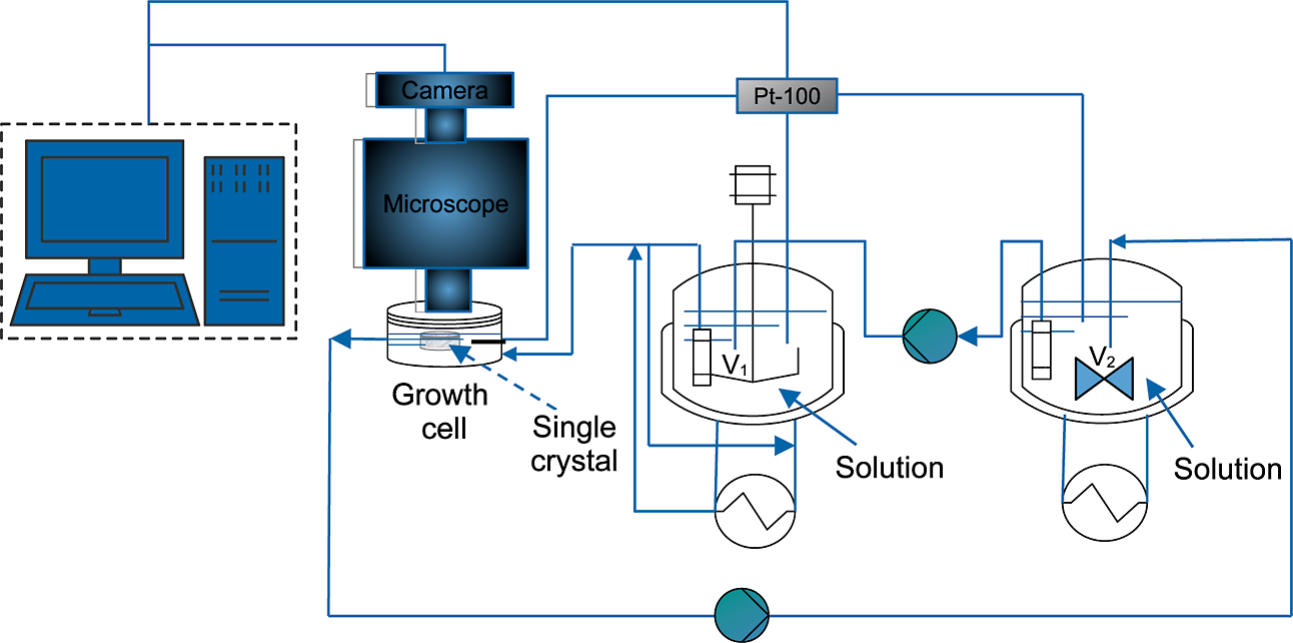
Schematic diagram of the experimental setup for single-crystal growth of boric acid. Modified from refs (42, 43). Reprinted (adapted) with permission from Gou, L.; Lorenz, H.; Seidel-Morgenstern, A. Investigation of a Chiral Additive Used in Preferential Crystallization. Cryst. Growth Des. 2012, 12 (11), 5197–5202. 10.1021/cg300042q. Copyright 2023 American Chemical Society.

The crystal growth of boric acid was studied in a single crystal growth cell. Figure 1 shows the experimental setup.^42,43^ The system integrates the double-jacketed growth cell and two vessels (*V*_1_ and *V*_2_). The cell is placed under a microscope (type Stemi2000C, company Carl Zeiss) with a mounted camera. The temperature of the components is regulated by thermostats using water.

A single crystal approximately 2 mm in size was glued on the pin head of the cells’ crystal holder, and a supersaturated solution was pumped through the cell at 4.7 mL/min using a peristaltic pump. The supersaturated solution was prepared using the vessels *V*_1_ and *V*_2_. In *V*_2_ was stored a saturated solution at 20 °C. It was pumped to *V*_1_ that was thermostated at 19 °C to get a relative supersaturation, σ, of 0.02, before entering the cell. This subcooling is sufficient to promote growth and minimize nucleation. Images were captured every 20 min for 8 h by the camera.

The aqueous solutions saturated with boric acid at 20 °C were prepared according to the solubility data reported in refs (14) and (44). Boric acid was dissolved in deionized water inside a double-jacketed thermostated vessel by stirring the solution until the measured refractive index verified the saturation. The supersaturation was calculated by eq 1
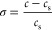
1where *c* is the solution concentration and *c*_s_ is the solute solubility. This equation is used throughout the article to estimate the supersaturation.

#### 2.3.1 Growth Dynamics and Rate Estimation

To determine the face dynamics during the growth process, the (001) face perimeter was chosen as a region of interest (ROI) from each figure, using the software Fiji.^45^ The ROIs were overlapped at the reference point given by the center of the crystal holder; after that, the axis coordinates were acquired and plotted with Python 3.9.^46^

**Figure 2:**
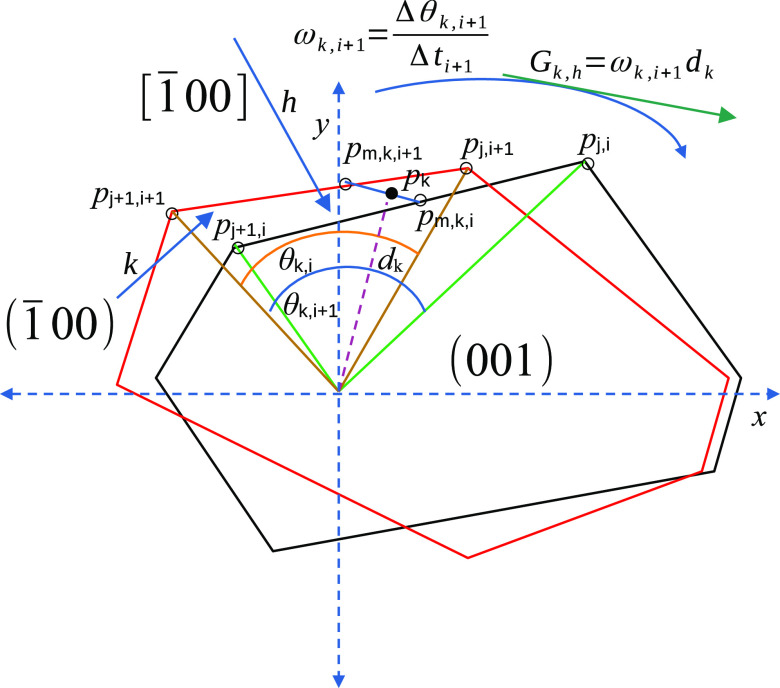
Scheme of the estimation of the growth rate for the (001) face from a supersaturated solution at σ = 0.02 and 19 °C, using the optical images acquired in the single crystal growth cell.

To estimate the growth rate, the following procedure was performed: (1) the angle θ_k_ between the borders (*p*_j_ and *p*_j+1_) of every edge *k* was measured for every time *t*_i_, (2) the angle difference was estimated between consecutive times Δθ_k,i+1_ = θ_k,i+1_ – θ_k,i_, (3) the angular velocity ω_i+1_ was calculated by dividing Δθ_k,i+1_ by Δ*t*_i+1_ = *t*_i+1_ – *t*_i_, (4) the middle point *p*_m,k,i_ over the line that connects the points *p*_j_ and *p*_j+1_ was calculated at *t*_i._ (this step was repeated for *t*_i+1_ to estimate *p*_m,k,i+1_), (5) the middle point *p*_k_ between the *p*_m,k.i_ and *p*_m,k.i+1_ was calculated, (6) the distance *d*_k_ between the origin coordinates and *p*_k_ was estimated, and (7) the angular velocity was multiplied by *d*_k_ to estimate the growth rate *G*_k_ of the face *k* in direction *h*. An illustrative scheme is presented in Figure 2. The numerical calculations and figures presented in this study were performed with Python 3.9.^46^

### 2.4 Atomic Force Microscopy Crystal Growth Measurements

**Figure 3:**
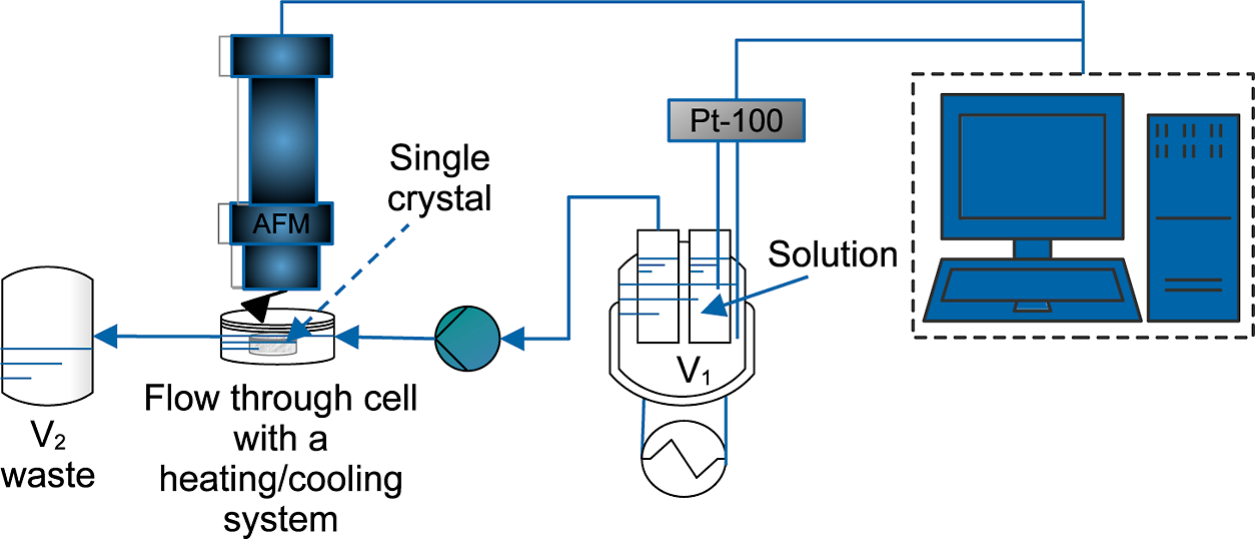
Setup of the AFM open flow-through cell for crystal growth measurement.

An Agilent 5500 AFM/SPM microscope with an open flow-through fluid cell of 0.5 cm^3^ and heating/cooling system was used to follow the surface changes of a single crystal of boric acid during growth. The heating/cooling system was composed by a Lakeshore 332 temperature controller together with the Peltier Cold MAC sample plate with water cooling; on the sample plate, the flow-through fluid cell was mounted. The cell was held at 23 °C, and surface scans were acquired in the contact mode. An uncoated silicon cantilever with a force constant of 0.2 N/m was applied. Figure 3 shows the experimental setup.

For each experiment, one single crystal of boric acid was glued on a glass slide, using two parts glue to expose the (001) face, and put it inside the flow cell. The cell was filled by pumping the saturated solution through it at 0.85 mL/min; after that, the flow rate was changed to 0.07 mL/min during growth. The solutions were not recirculated. To perform the measurement for the next concentration, a fresh solution was pumped through the cell at 0.85 mL/min. Scans were acquired every 64 s, and the scanned area was 20 × 20 μm^2^. The bulk supersaturation (σ = 0.07) was generated by cooling down a saturated solution of boric acid at 25 °C in a thermostatic vessel. This subcooling was sufficient to promote growth and minimize nucleation. To choose the surface of the study, a crystal surface of 90 × 90 μm^2^ area was scanned in air, then a 20 × 20 μm^2^ area of that surface was scanned to verify the presence of characteristics points (terraces, steps, edges, and dislocations) and the highest point was less than 4 μm (height limit of the scanner).

The saturated solutions were prepared in glass jars, performing for every sample the following procedure: (1) The substances were massed inside a glass jar in the order: boric acid, salt and water, (2) a stir bar was put inside the glass jar and shaked in a vortex to avoid the packing of the solids, (3) the sample prepared was kept inside a vessel thermostated at 25°C by a thermostatic bath, and stirred at 700 rpm with a magnetic stirrer until equilibrium was reached, it was verified measuring the refractive index,^14,15^ (4) each sample was filtrated using a syringe filter (0.45μm) and put the filtered solution inside other glass jar and kept in a vessel thermostatted at 25 °C. Boric acid saturated solutions with different concentrations of Na_2_SO_4_^15^ or Li_2_SO_4_^14^ were prepared. The concentrations of the salts in the solutions were 0, 1, 5, and 16 wt % mass for Na_2_SO_4_ and 1, 5 and 8 wt % mass for Li_2_SO_4_.

#### 2.4.1 Growth Dynamics and Rate Estimation from AFM Scans

The growth dynamics was determined by tracking the advancement of steps for the layers marked in the AFM figure (e.g., Figure 9). This figure shows the position of the previously marked layers in time *t* and the growth directions they advance to reach the positions described in *t*_i+1_. This information was used to estimate the velocity of the advancement of steps, *v*. The procedure to calculate the velocity is described as follows: (1) the distance (radius) and angle to each edge (growth direction) from the same reference point and their change in time were followed; (2) the step advance, *d*_j_, was estimated by the distance between the points (*r*_i_,θ_i_) and (*r*_i+1_,θ_i+1_) at the edges at time *t*_i_ and *t*_i+1_ by eq 2
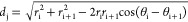
2

(3) the value of *v* was calculated by dividing *d*_j_ by the time difference Δ*t* = *t*_i+1_ – *t*_i_. All AFM scans in this study were analyzed using the software Gwyddion,^101^ and the numerical calculations and figures presented were performed with Python 3.9.^46^

### 2.5 Boric Acid Crystal Morphology Prediction

Boric acid crystal morphology was predicted with WinXMorph.^47,48^ The parameters used were *a* = 7.05 Å, *b* = 7.05 Å, *c* = 6.57 Å, α = 92.5°, β = 101.17°, and γ = 120°, and the symmetry/point groups was 1̅. The unit cell and structural parameters were taken from Zachariasen.^49^

### 2.6 Boric Acid Unit Cell

The boric acid unit cell was created with VESTA 3.^50^ The parameters used were single unit cell = triclinic, radii type atomic, central atom B, isosurfaces *F*_min_ = −28.4059 and *F*_max_ = 354.258. The unit cell and structural parameters were taken from Zachariasen.^49^

## 3 Results and Discussion

### 3.1 Crystal Growth Studies in the Single-Crystal Growth Cell

**Figure 4:**
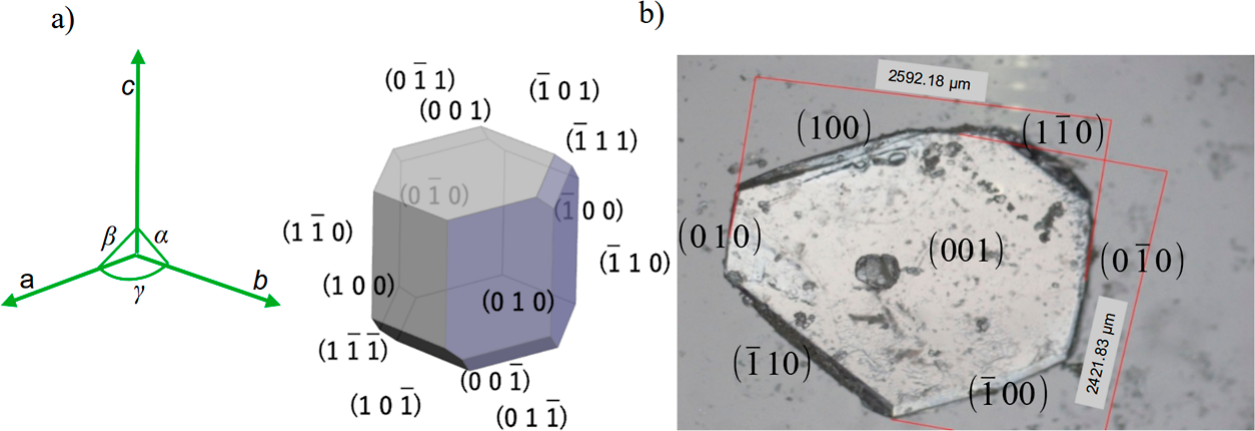
(a) Boric acid crystal morphology predicted with WinXMorph^47,48^ from the crystalline structure reported by Zachariasen.^49^ (b) Single crystal of boric acid grown by evaporation from aqueous boric acid solutions saturated at 20 °C.

Orthoboric acid (H_3_BO_3_) is a layered material parallel to the basal plane of a triclinic crystal structure. The triclinic unit cell, containing four molecules of B(OH)_3_, has the dimensions *a* = 0.7039 nm, *b* = 0.7053 nm, *c* = 0.6578 nm, α = 92.58°, β = 101.17°, and γ = 119.83°.^51^ In each layer, one boron atom is surrounded by three oxygen atoms to form a triangular BO_3_ group. Hydrogen bonds link the BO_3_ planar groups together to form endless layers of nearly hexagonal symmetry.^51^ The electronegativity of boron and oxygen is 2 and 3.5, respectively; thus, the bonding between them is described as mostly covalent with some ionic character.^52^ The bond distances within a B(OH)_3_ molecule are B–O = 0.136 nm and O–H = 0.088 nm, with a value of 114° for the oxygen bond angle. The O–H··O distance between molecules is 0.270 nm. The layers are 0.318 nm apart and are held together by weak van der Waals forces.^49^ The volume of the unit cell is 0.263 nm^3^; it contains four boric acid molecules.^52^ Based on the boric acid crystal structure reported by Zachariasen,^49^ the crystal morphology was predicted with WinXMorph.^47,48^ It is shown in Figure 4a. The main faces present are (001), (010), (100), , , , and . The small faces , , , ,  and  are also present. The (001) face is the basal plane. The morphology was used to identify the crystal faces and edges present in the single crystals grown by evaporation from boric acid solutions saturated at 20 °C. The identified features are depicted in Figure 4b. It shows the presence of the (001) face and the edges of the (010), (100), , , , and  faces.

**Figure 5:**
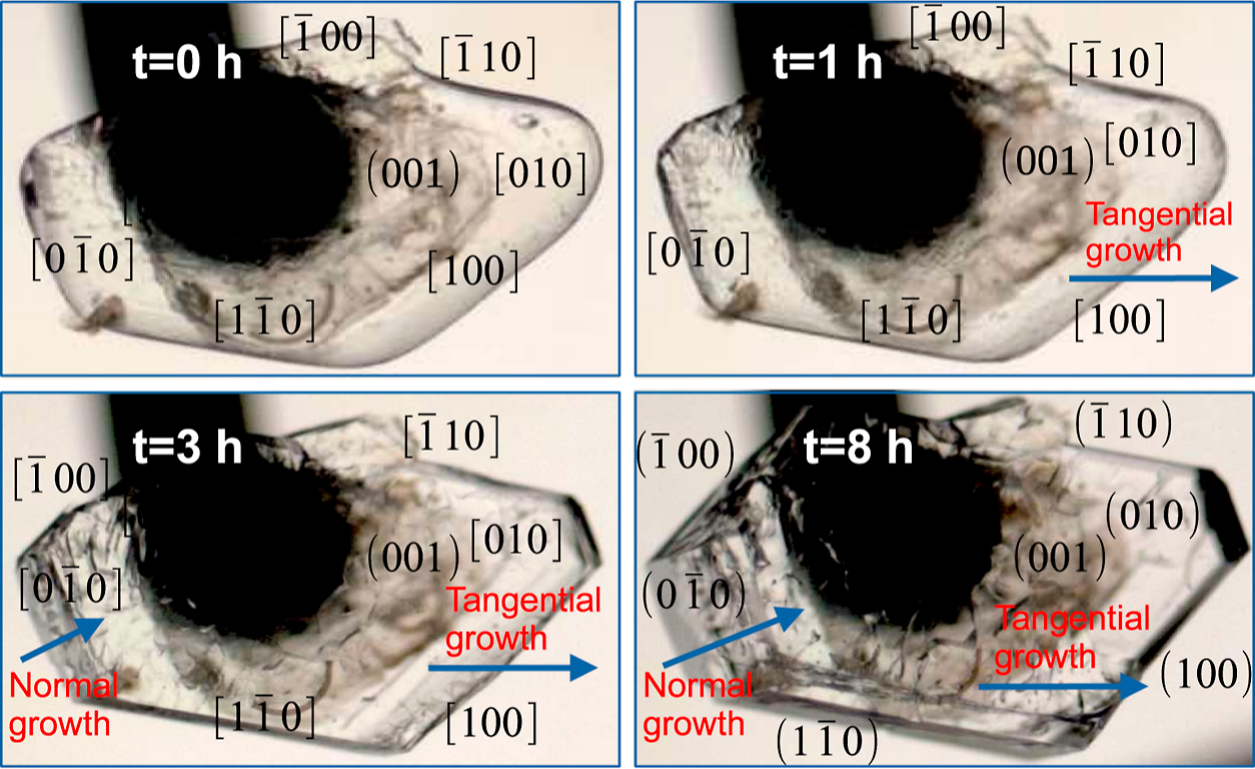
Single crystal growth of boric acid from a supersaturated solution at σ = 0.02 and 19 °C.

Figure 5 shows the observed crystal growth of boric acid.

**Figure 6:**
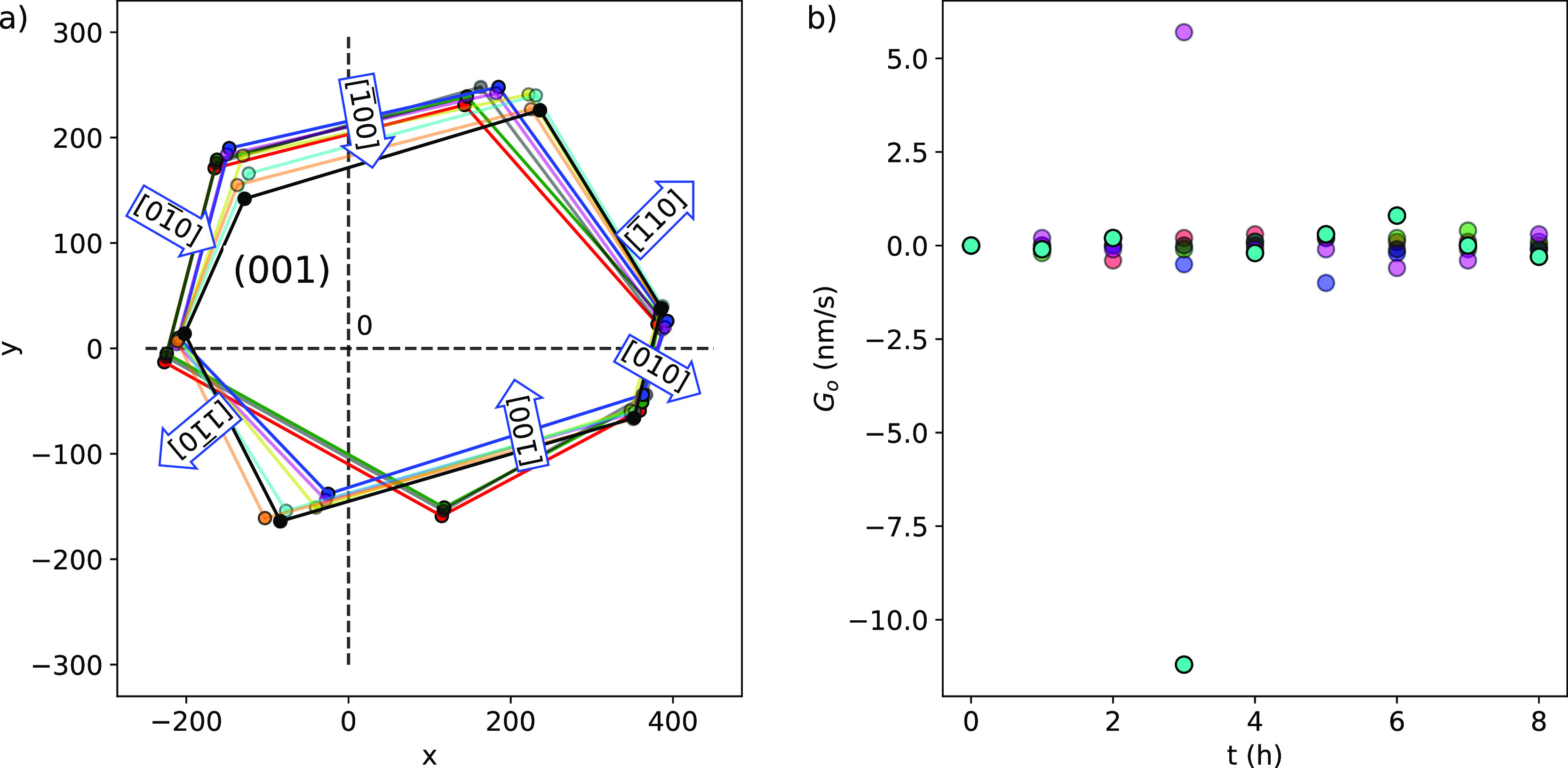
(a) Growth dynamics for the (001) face growth of boric acid from a supersaturated solution at σ = 0.02 and 19 °C. Time *t*: -●- (red), 0 h; -●- (green), 1 h; -●- (gray), 2; -●- (blue), 3 h; -●- (purple), 4 h; -●- (yellow), 5 h; -●- (sky blue), 6 h; -●- (golden), 7 h; and -●- (black), 8 h. (b) Resulting growth rate for the (001) face growth of boric acid in the growth directions: ● (red), [010]; ● (blue), ; ● (light green), ; ● (dark green), ; ● (purple), ; -●- (sky blue), [100].

Figure 6 shows that the (001) face grew in all directions with variable rates that change dynamically, having maximum values at different times (see Table 1).

**Table 1:** Maximum, *G*_max_, and Average, *G*_avg_, Growth Rates of the (001) Face of Boric Acid Grown from Its Aqueous Solutions at 19 °C and Supersaturation σ = 0.02

growth direction	*G*_max_ [nm/s]^a^	*G*_avg_ [nm/s]^a^
[010]	–0.4 (*t* = 2 h)	0.07 ± 0.2
[1̅10]	–1.0 (*t* = 5 h)	–0.2 ± 0.3
[1̅00]	0.4 (*t* = 7 h)	0.1 ± 0.2
[01̅0]	0.2 (*t* = 5 h)	–0.01 ± 0.09
[11̅0]	5.7 (*t* = 3 h)	0.54 ± 1.84
[100]	–11.2 (*t* = 3 h)	–1.17 ± 3.56

a The negative sign indicates that the steps advance in the opposite direction to the measurement lines.

The variation in growth rates of the edges is the most significant at or after 3 h, except for the [010] direction, which exhibits the maximum growth rate at 2 h. The more favorable growth directions to complete the growth of each layer are [100] and . The growth in the [100] direction is the fastest. The average growth rates decrease in the order [100] >  >  >  > [010] > . Moreover, it was observed that the growth faces describe a rolling mechanism,^53^ where uncurled edges [, , and (010)] and curled edges [, (100), and ] coexist at the layers. The rotation is more pronounced for the directions , , and ; thus, their edge widths decrease. That behavior is opposite to that observed for the edges  and (100), whose sizes increase, and (010), which is not significantly modified. It could explain the tangential growth observed for the (001) face in Figure 5. The average normal growth rate calculated was 34.6 nm/s for the (001) face. To verify the consistency of the calculated growth rates, they were compared with the overall growth rate for boric acid, 1.4 × 10^–8^ m/s (14 nm/s) at 20 °C, estimated from measurements in a fluidized bed.^18^ It shows that both normal and tangential growth rates are consistent. The closer value to the literature is the maximum *G* in the [100] direction. The normal growth rate is 2.5 times greater than the literature value. It can be explained by assuming a spherical shape for boric acid crystals in ref (18) to estimate the mass growth rate *R*_G_ and afterward convert it to *G*. Also, hydrodynamic effects could be involved. Therefore, it was determined that the boric acid crystal grows from its aqueous solution by spiral growth. The fastest growth direction for the face (001) is [100]. The rotation is more pronounced for directions  and , decreasing their edge widths. The growth rates estimated are consistent. The growth dynamics agrees with the growth mechanisms reported in ref (53) for the synthesis of ultra-long one-dimensional boric acid microstructures that were characterized by transmission electronic microscopy (TEM).

### 3.2 Crystal Growth Monitoring by AFM

#### 3.2.1 Surface Structure

**Figure 7:**
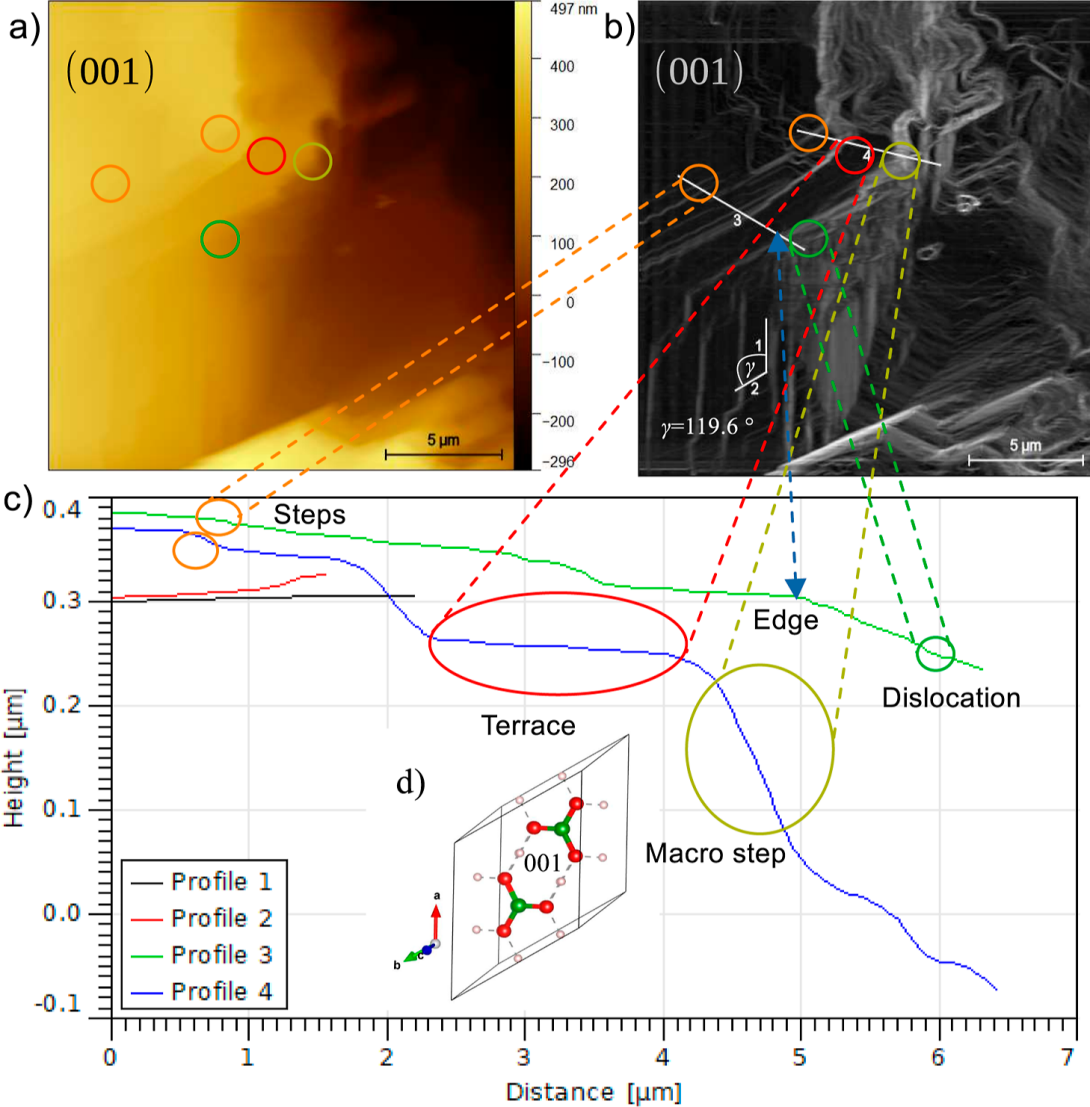
(a) Topography of the (001) face of a single crystal of boric acid taken by AFM in air at room temperature. (b) Step edges and (c) profiles obtained from the AFM image given in (a). (d) Unit cell of boric acid elaborated with VESTA 3^50^ using the crystalline structure reported by Zachariasen.^49^ For the boric acid molecule, the boron, oxygen, and hydrogen atoms are represented by green, red, and light red colors, respectively.

The AFM image of a boric acid crystal acquired in air is depicted in Figure 7a. This image was used to obtain the step edges (Figure 7b) and to determine the height profiles of characteristics regions of the crystal surface (Figure 7c). The profiles together with the boric acid unit cell (Figure 7d) created with VESTA 3^50^ from the crystalline structure reported by Zachariasen^49^ were used to identify the surface characteristic features, and their crystal face corresponds to the exposed surface on the AFM images. The measurement of the heights for profiles 3 and 4 confirmed that the surface is composed by terraces, dislocations, edges, steps, and macrosteps (see Figure 7c). Edges and dislocations are relevant because they are favorable sites for the integration of the growth units from the solution.

The terraces, steps, and macrosteps are also important to determine the growth mechanism and rate. Also, the heights of profiles 3 and 4 show that the steps and macrosteps are composed of multilayer boric acid molecules (*c* = 0.6578 nm and layer separation = 0.318 nm).

The angle between the edges of profiles 1 and 2 (γ = 121°) supports that the (001) face is exposed because that value agrees with the angle γ = 119.61° between the *a* and *b* axes of the unit cell (see Figure 4).

#### 3.2.2 Crystal Growth of Boric Acid

**Figure 8:**
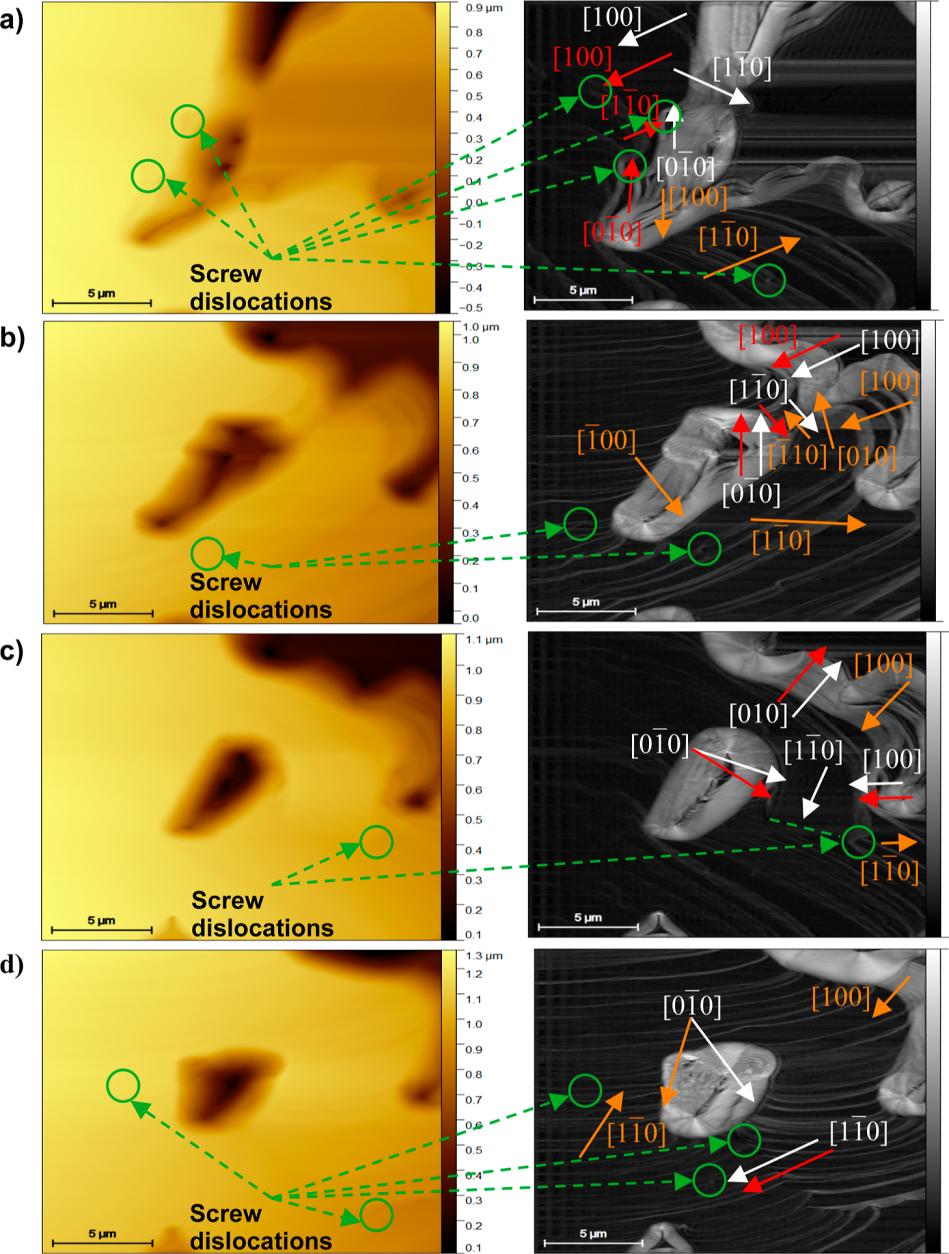
Evolution of steps on the (001) face of boric acid grown from its aqueous solutions at 23 °C and supersaturation σ = 0.07. Topography (gold color) and edges (gray color) at time *t*: (a) 8.01, (b) 9.05, (c) 12.17, and (d) 13.21 min.

**Figure 9:**
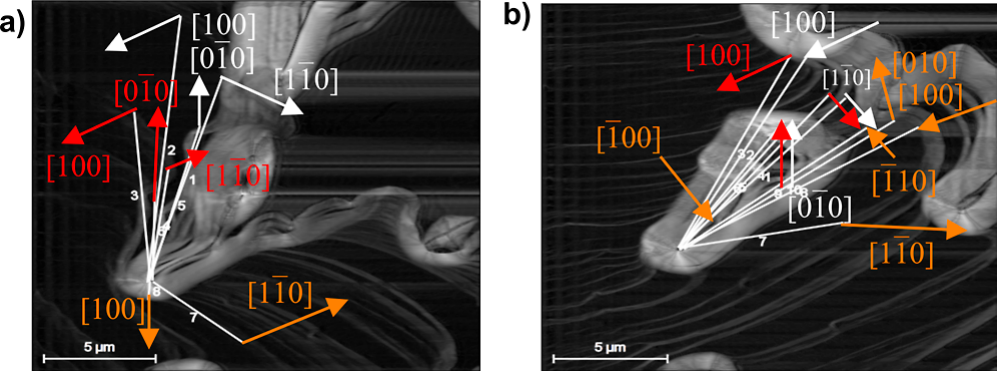
Tracking of the steps’ edges on the (001) face of boric acid grown from its aqueous solutions at 23 °C and at σ = 0.07. Time *t*: (a) 8.01 and (b) 9.05 min.

The crystal growth of boric acid was analyzed following the evolution of steps on the (001) face of crystals grown from its aqueous solutions at 23 °C and supersaturation σ = 0.07. The images of the topography (gold color) captured and the steps’ edges (gray color) derived from them are shown in Figure 8. The time frames considered were: (a) 8.01, (b) 9.05, (c) 12.17, and (d) 13.21 min. The AFM scans show that boric acid crystal growth is multilayered and occurs by a rolling mechanism and that sources of steps for growing are dislocations on the surface (see Figure 8). This behavior was revealed by analyzing the changes of the steps’ edges over time. The multilayer growth was observed 8.01 min after the crystal growth started. To determine the growth directions of the layers over the (001) face, three layers were controlled. The layers were marked by arrows of white, red, and orange colors at the edges’ images in Figure 9. The growth directions were identified based on the unit cell orientation (Figure 7d) and the crystal morphology (Figure 4).

**Figure 10:**
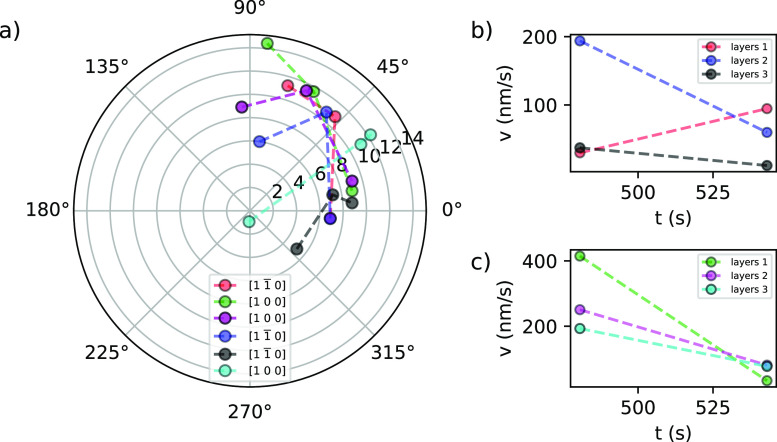
(a) Advancement of steps’ edges on the (001) face of boric acid grown from its aqueous solutions at 23 °C and supersaturation σ = 0.07. Point changes are given for time increase from 8.01 to 12.17 min. (b) Velocity of advancement of steps *v* in the  direction. (c) Velocity of advancement of steps *v* in the [100] direction.

Figure 10a shows the changes of position of the layers due to their movements in different growth directions for the time frame from 8.01 to 12.17 min.

The values for the fastest growth directions are presented in Figure 10b,c. The average velocity of the advancement of steps *v* on the (001) face in the  direction is 71.17 ± 42.30 and in the [100] direction is 174.83 ± 36.96 [nm/s]. The fastest growth is in the [100] direction.

Figure 8 shows that the edge  advances in the clockwise direction, while  advances in the opposite direction. The presence of the growth directions [100], , [010], and  were also observed. The edge advancement has the same behavior as described by the single-crystal growth cell measurements in Figure 6a. The screw dislocations are the sources of steps for the incorporation of growth units on the crystal surface. Therefore, the mechanisms of growth of boric acid at σ = 0.07 (Δ*T* = 2 K) and 23 °C is derived to be a spiral growth driven by screw dislocation. It implies that molecules of boric acid diffuse from the solution to the actives sites at the steps present in dislocations, where they are integrated, thus making the step advancement to complete a crystal face to grow the crystal in the normal direction. The growth mechanism determined agrees with that inferred from the growth cell and that reported in the literature.^53^ Moreover, the results give further insights into the growth dynamics.

**Figure 11:**
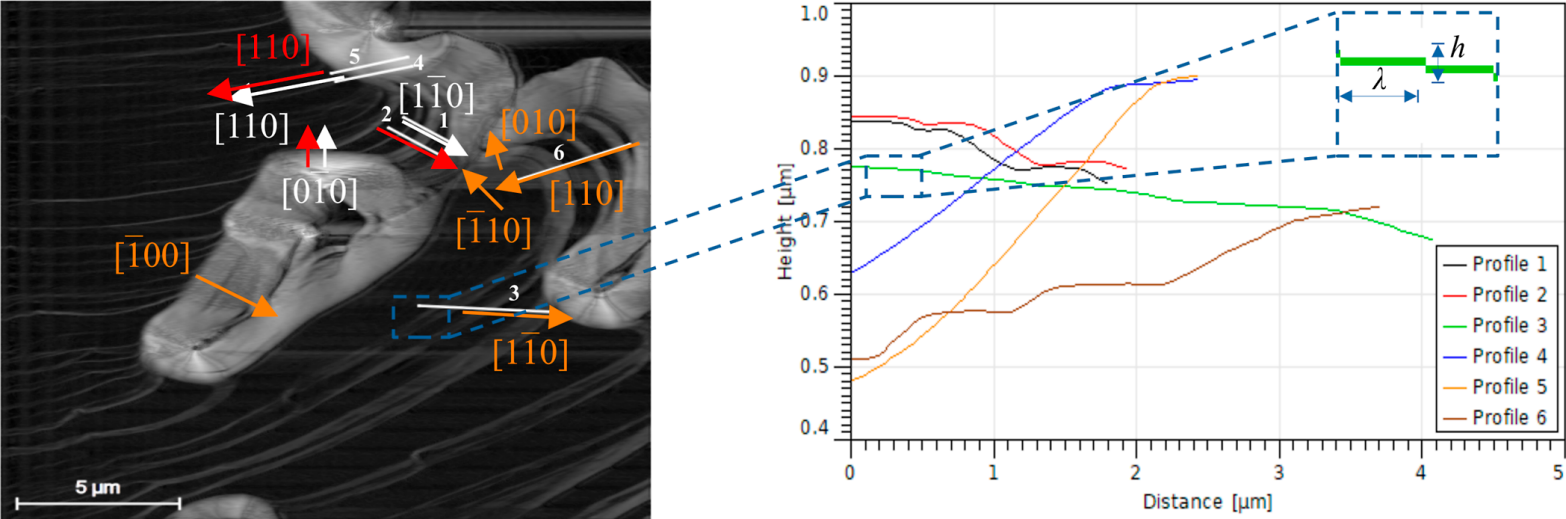
Tracking of the width of the terraces λ and height of the steps *h* on the (001) face of boric acid during growth from its aqueous solutions at 23 °C and supersaturation σ = 0.07, at time *t* = 9.05 min.

For the spiral growth mechanism with a single source, the normal growth rate and velocity of advancement of steps are related by the expression^54^
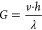
3where *h* is the step height and λ is the step distance after a spiral turn is complete. These features were estimated from the AFM scans at *t* = 9.05 min (see Figure 11).

They were considered as the average values. *G* was calculated by multiplying the *v* in the [100] direction, the fastest velocity, by its corresponding ratio *h*/λ. The calculated value is *G* = 10.29 ± 3.54 nm/s. The consistency of this value was determined by comparison with the overall growth rate *G* = 3.7 × 10^–8^ m/s (37 nm/s), extrapolated from the measurements in a fluidized bed.^18^ Although the value of the (001) face is lower than the reference, it is consistent. The difference in values may be attributed to hydrodynamic effects, the reference value considers all crystal faces growth, and the growth surface is given by multiple spirals.

The edge-free energy for the (001) face can be estimated by the expression
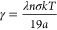
4

It was derived from the equation given in ref (55), where *k* is the Boltzmann constant, *T* is the crystallization temperature, *n* is the amount of cooperating spirals, and  is the specific molar volume units. For *n* = 1, the edge (100) γ is 0.047 J/m^2^ and γ is 0.054 J/m^2^ for . Assuming that the supersaturation is isotropic, the γ values show that the growth unit integration is more favorable at the edge (100) than for ; also, spirals are not circular; therefore, the average γ can be calculated using the average λ.^56^ Considering the average λ = 240 nm for the fastest growth directions ([100] and ), γ is 0.051 J/m^2^.

### 3.3 Growth of Boric Acid in the Presence of Impurities

**Figure 12:**
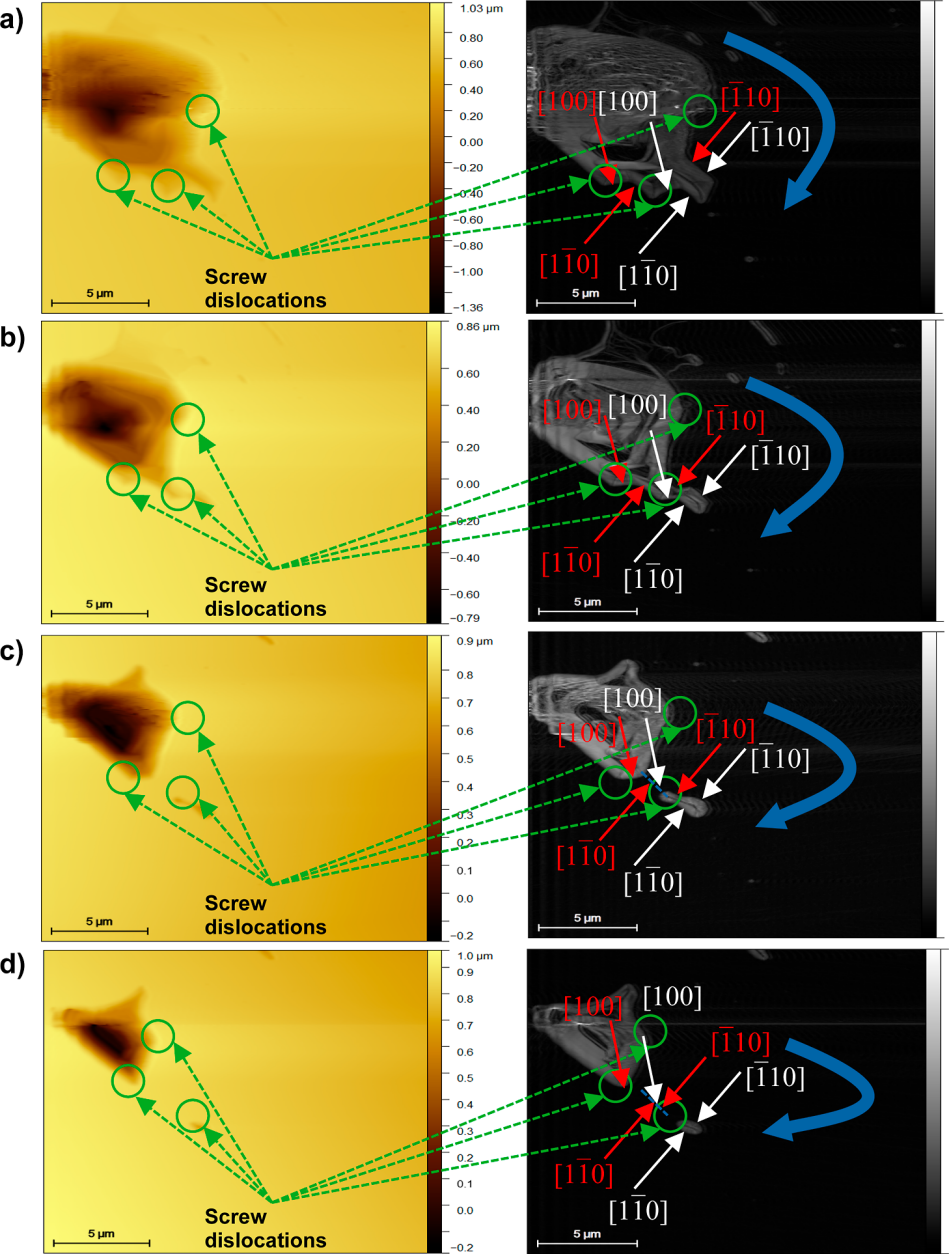
Evolution of steps on the (001) face of boric acid grown from its aqueous solutions in the presence of sodium sulfate (5 wt %) at 23 °C and supersaturation σ = 0.07. Topography (gold color) and edges (gray color) for time intervals of 64 s (a–d).

**Figure 13:**
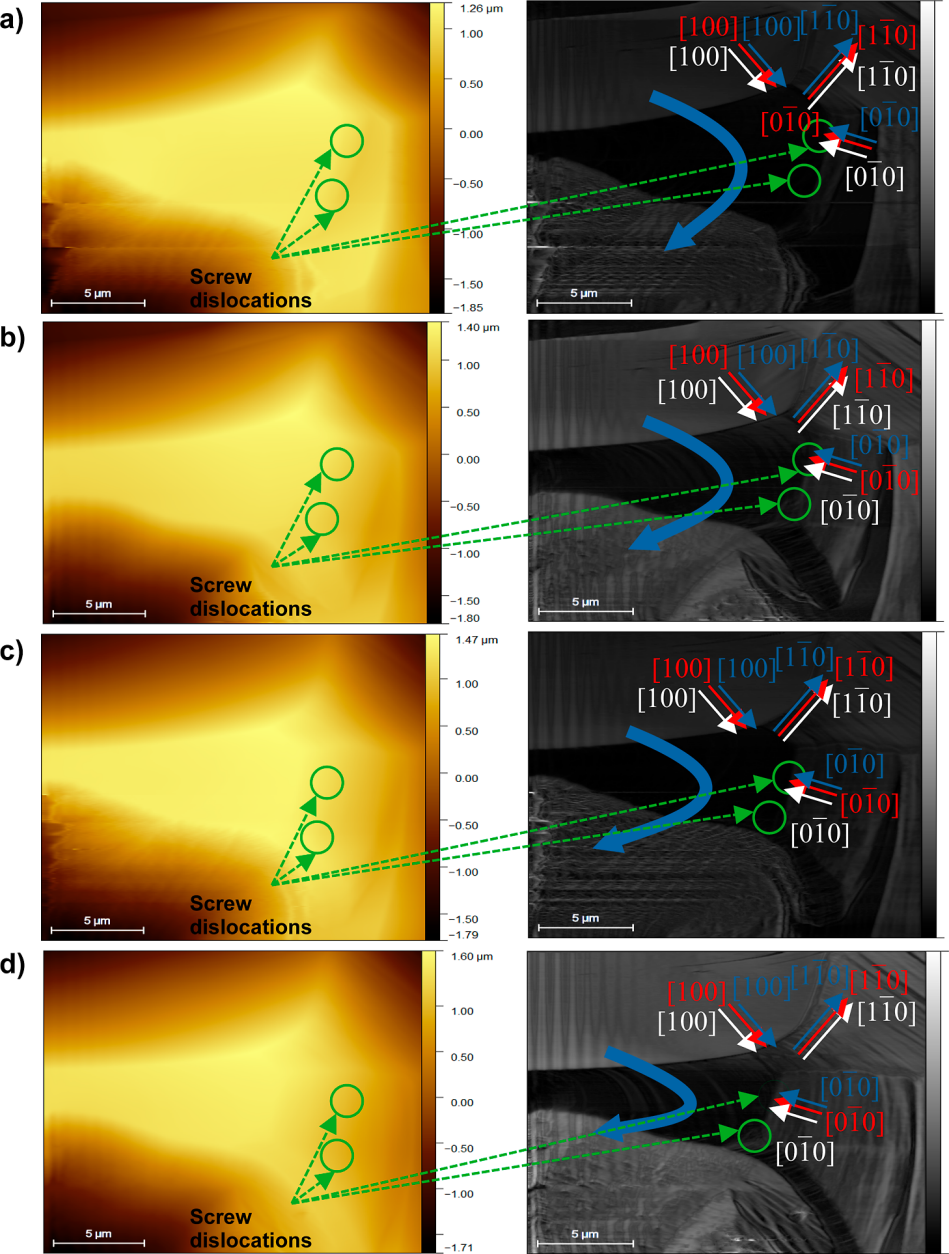
Evolution of steps on the (001) face of boric acid grown from its aqueous solutions in the presence of lithium sulfate (5 wt %) at 23 °C and supersaturation σ = 0.07. Topography (gold color) and edges (gray color) for time intervals of 64 s (a–d).

**Figure 14:**
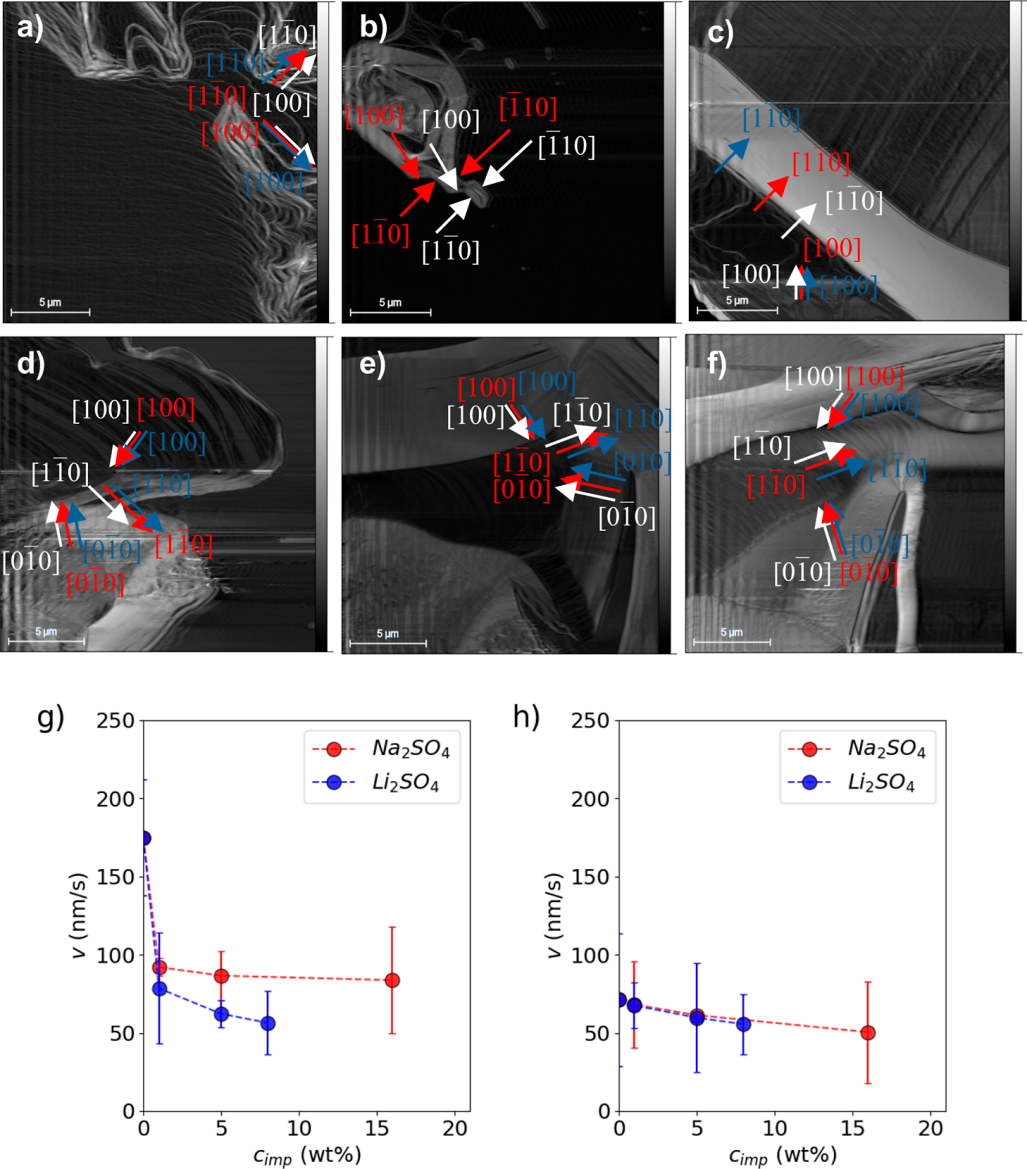
Advancement of steps on the (001) face of boric acid at 23 °C and supersaturation σ = 0.07 at different salt concentrations *c*_imp_ of sodium sulfate: (a) 1, (b) 5, and (c) 16 wt %, and lithium sulfate at (d) 1, (e) 5 and (f) 8 wt %, and average velocity of advance of steps *v* of the (001) face in the directions (g) [100] and (h)  at the same salt concentrations, supersaturation, and temperature stated above. ---, trend lines. —, experimental bar lines.

The effect of impurities on the crystal growth of boric acid was determined by tracking the advancement of steps on the (001) face and the presence of dislocations on the crystal surface (see Figures 12–14) in the absence and presence of sodium sulfate or lithium sulfate at 23 °C and σ = 0.07. To show clearly the changes on the crystal surface, the surface edges were determined from the AFM images. In the presence of sodium sulfate, the advancements of the steps for growth directions [100], , , and  were identified (Figures 12 and 14).

The evolution of the surface growth shows a spiral growth mechanism from screw dislocation sources as demonstrated in Figure 12. The same mechanism was determined in the presence of lithium sulfate (see Figure 13). In both cases, the steps advance in the same directions as found for the acid surface grown from boric acid aqueous solutions.

Figure 14a–f shows that the presence of dislocations on the crystal surface decreases with concentration increments of sodium and lithium sulfate; therefore, the surface becomes soft. The presence of dislocations affects the advancement of the steps for all growth directions identified ([100], , , and ). The inhibition of the step advancements could be explained by the salt adsorption on the steps. To determine which salt and in which concentration have the greatest effect on the surface growth, the velocities of advancement of steps in directions [100] and  were estimated from the edges of the AFM images using the procedure described in Section 2.4.1. The velocity was calculated in those directions because they were identified in all AFM images. The average velocity, *v*, values determined are presented in Figure 14g,h.

**Figure 15:**
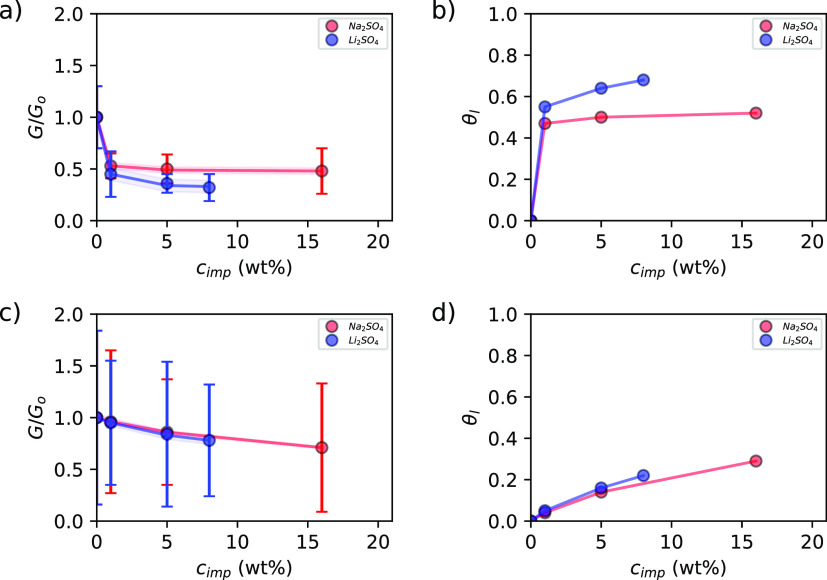
Relative growth rate *G*/*G*_o_ of boric acid in the presence of, *c*_imp_, in wt % of Na_2_SO_4_, ●(red), and Li_2_SO_4_, ●(blue), at 23 °C and σ = 0.07.—, calculated from eq S7 for the (001) face in directions: (a) [100] and (c) . The coverage of active sites by an impurity, θ_l_, for the boric acid crystal surface in the presence of, *c*_imp_, Na_2_SO_4_, ●(red), and Li_2_SO_4_, ●(blue), —, calculated from eq S2 at 23 °C and σ = 0.07 for the (001) face in directions: (b) [100] and (d) . Shaded regions indicate 95% confidence intervals for the model forecast. —, experimental bar lines.

In direction [100], the velocity decreases strongly with salt concentration increments, even at the lowest salt concentration of 1 wt %. Although the velocity is lower in the presence of lithium sulfate compared to sodium sulfate, the velocity error bars show no difference between the effects of the two salts. Moreover, trend lines verify that this behavior is kept at higher salt concentrations. In contrast, for the direction , the velocity slightly decreases with the increasing salt concentration. Also, in this case, no difference of the salt effects is seen. A further insight of the salt effect on the crystal growth can be derived from the normal relative growth rate, *G*/*G*_o_. It was estimated from the relative advancement of steps, *v*/*v*_o_, because they are proportional.^57^ This ratio is calculated from the velocity of advancement of steps without, *v*_o_, and in the presence of salts, *v*. The values estimated are presented in Figure 15a,c.

Moreover, the salt adsorption isotherms on the growth surface can be estimated from the competitive adsorption model (CAM)^11^ (see the Supporting Information). This is applied to estimate the isotherms because they cannot be measured by classical methods. This is due to the fact that when boric acid crystals are stirred in sodium sulfate aqueous solutions at a constant temperature, the crystals are dissolved, because boric acid solubility is increased in the presence of sodium sulfate.^58^ This behavior is opposite in the presence of lithium sulfate and boric acid precipitates, because the acid solubility is decreased in the presence of this salt.^14,15^ Also, the classical methods measure the adsorption over the whole solid surface, not on specific crystal faces.

The topography of the surface in the presence of these impurities suggests that the inhibition of the growth rate is due to their adsorption on the crystal surface (see Figures 14 and 15b,d). It is dependent on the growth direction (surface edge). The salt adsorption is higher at the (100) edge and at lower salt concentrations than for . It indicates that the salt effect is different for every edge. To determine the impurity effect mechanism, molecular modeling simulations^57^ or theoretical models can be applied. In this study, the CAM model was used to explain the AFM observations. The results are presented in Figure 15 and in the following paragraphs.

For the *G*/*G*_o_ estimated from the velocities in the [100] growth direction, the values of the parameters of the model are *k*_Na_2_SO_4__ = 9.62 ± 1.94 (100 g solution/g solute) and β_Na_2_SO_4__ = 0.52 ± 0.01 and *k*_Li_2_SO_4__ = 3.91 ± 0.70 (100 g solution/g solute) and β_Li_2_SO_4__ = 0.69 ± 0.01, respectively. The standard error of estimation (S.E.) and coefficient of determination *R*^2^ are 0.01 and 0.9995, respectively, in the presence of sodium sulfate; S.E. is 0.01 and *R*^2^ is 0.9987 in the presence of lithium sulfate. The parameter *k* equals zero in both cases. The parameters were estimated using the software Gretl.^59^ The results show that for the (001) face, the impurities adsorbed extensively on the crystal surface (*k*_Na_2_SO_4__ > *k*_Li_2_SO_4__ > 1) and that the affinity of the salts for actives sites is greater for lithium sulfate than for sodium sulfate (β_Li_2_SO_4__ > β_Na_2_SO_4__) at the (100) edge, a fact that explains the greater effect of Li_2_SO_4_ than Na_2_SO_4_ on the growth rate of boric acid (see Figure 15a,b). The values of the parameters presented are statistically significant with a confidence level equal to or greater than 0.95.

From the *G*/*G*_o_ estimated from the *v* in the  growth direction, the values of the parameters of the model are *k*_Na_2_SO_4__ = 0.07 ± 0.01 (100 g solution/g solute) and β_Na_2_SO_4__ = 0.55 ± 0.03 and *k*_Li_2_SO_4__ = 0.11 ± 0.02 (100 g solution/g solute) and β_Li_2_SO_4__ = 0.48 ± 0.07, respectively. Also, in this case, *k* equals zero for every salt. In the presence of sodium sulfate, S.E. and *R*^2^ are 0.003 and 0.9995, respectively; S.E. is 0.01 and *R*^2^ is 0.9956 in the presence of lithium sulfate. The parameters for sodium sulfate and lithium sulfate are statistically significant with a confidence level equal to or greater than 0.95. The analysis of the results reveals that salt adsorption is in the same range as that for [100] (0.40 < β_i_ < 0.70) and slightly greater for lithium sulfate than for sodium sulfate because the value of k_Na_2_SO_4__ is slightly smaller than for k_Li_2_SO_4__ and that salt adsorption on the crystal surface is low (*k*_i_ < 1) and β_Na_2_SO_4__ is equal to β_Li_2_SO_4__. This implies that competitive adsorption between these salts and boric acid molecules for active sites at the  edge is low; for those reasons, the inhibition of growth rate is low (see Figure 15c,d).

Therefore, the effect of sodium sulfate and lithium sulfate on the boric acid growth rate is anisotropic. At the (100) edge, the salts are extensively adsorbed (*k*_i_ > 1) and are moderately active impurities (β < 1) that decrease the boric acid growth rate. For the  edge, the adsorption of the salts from the solution to the crystal surface is more favorable than migration across the surface and incorporation at the active sites of the  edge (*k*_i_ ≪ 1 and β < 1). Both salts are adsorbed independent of the supersaturation (*k* = 0 and *k*_i_ > *k*). This signifies that boric acid growth rate is reduced but not completely inhibited in the presence of these salts and that the main adsorption of the impurities at the active sites over the crystal surface occurs at the (100) edge.

At concentrations greater than 5 wt %, for both impurities, some nuclei were formed inside the fluid cell. This might be a result of contact with the cantilever, vibration of the fluid by the peristaltic pump, or crystallization at the tip of the cantilever (radius is under 10 nm).

## 4 Conclusions

The real-time monitoring of the crystal growth of boric acid from an aqueous boric acid solution in the absence and presence of sodium sulfate or lithium sulfate by AFM in situ, to determine the effect of these impurities on the boric acid growth mechanism and relative growth rate, was successfully implemented. Also, the investigation of the boric acid growth in a single-crystal growth cell to support the AFM study was successfully performed.

The results obtained from the single-crystal growth cell showed that boric acid grows layer by layer. The cleavage face is the (001) one. Boric acid grows more favorably in directions  and [100] to complete the growth of each layer. The growth direction [100] is the fastest. Moreover, the perpendicular growth is greater than tangential growth for the (001) face, which could be explained for by the spiral growth of the layers.

The AFM in situ measurements revealed that the growth of the (001) face of a boric acid crystal from aqueous solutions is controlled by spiral growth driven by screw dislocations in the absence and presence of sodium and lithium sulfate and that these salts reduce the relative growth rate of boric acid but do not inhibit it completely. Moreover, the salt effects are anisotropic. At the (100) edge ofthe steps of the (001) face, the impurities are moderately active, adsorbing on the actives sites and inhibiting the formation of sources of steps such as dislocations, therefore, reducing the velocity of advancement of the steps, while at the  edge, the impurities are moderately active but not extensively adsorbed. For both salts, the adsorption on the crystal surface was independent of the supersaturation.

The findings can be useful for the improvement of the quality of boric acid recovered from brines and minerals and the synthesis of nanostructures and microstructures of boron-based materials.

## Supporting Information Available

Description of the competitive adsorption model (CAM) proposed by Martins et al.,^11^ used to explain the effect of impurities on the crystal growth rate (PDF)
