# Supplementary material for: Real-Time Crystal Growth Monitoring of Boric Acid from Sodium or Lithium Sulfate Containing Aqueous Solutions by Atomic Force Microscopy
Source: ACS Omega. 2023 Mar 20;8(12):10822–35. doi: 10.1021/acsomega.2c06953 (PMC10061539; doi:10.1021/acsomega.2c06953)
Supplement: Supplementary file 1 — ao2c06953_si_001.pdf [file ao2c06953_si_001.pdf]

Supporting Information

Real-time crystal growth monitoring of boric acid from sodium or lithium sulfate containing aqueous solutions by atomic force microscopy

Wilson Alavia,[*,†‡] Andreas Seidel-Morgenstern,[‡,§] Dana Hermsdorf,[‡] Heike Lorenz,[‡] Teófilo A. Graber[∥]

[†] Faculty of Engineering, Universidad Alberto Hurtado, Almirante Barroso 10, 8340575 Santiago, Chile.

[‡] Max Planck Institute for Dynamics of Complex Technical Systems Magdeburg, Sandtorstraße 1, D-39106 Magdeburg, Germany.

[§] Institute for Process Engineering, Otto von Guericke University Magdeburg, Universitätsplatz 2, D-39106 Magdeburg, Germany.

[∥]Departamento de Ingeniería Química y Procesos de Minerales, Universidad de Antofagasta, Antofagasta, 1270300, Chile.

Theoretical background

To explain the effect of impurities on the crystal growth rate Martins et al.[1] have proposed the Competitive Adsorption Model (CAM). It describes crystal growth in presence of impurities as a competition between solute molecules and impurities for adsorption on the actives sites on the crystal surface. The determinant factors of success of each species are related to its concentration, mobility and physicochemical affinity to the crystal surface. Also, this model assumes that solution properties are not changed by impurities and the system is in steady state. To represent the competition between solute molecules and impurities, the overall surface coverage, θ_s, is assumed to be described by an extended Langmuir isotherm:

$$\theta_s = \frac{K_i c_{imp}}{K_i c_{imp} + Kc + 1} \qquad \text{(S1)}$$

where c and c_{imp} are the solute and impurity concentrations in the solution. Representing this equation as a function of supersaturation, $\sigma=(c-c_s)/c_s$, is obtained:

$$\theta_s = \frac{k_i c_{imp}}{k_i c_{imp} + k\sigma + 1} \qquad \text{(S2)}$$

$$k_i = \frac{K_i}{1 + K c_s} \qquad \text{(S3)}$$

$$k = \frac{K c_s}{1 + K c_s} \qquad \text{(S4)}$$

with k_i and k, being the constants for the adsorption of solute and impurity respectively, and c_s, the solute solubility. For the crystal growth process from impure solutions the following steps are considered: 1) surface adsorption of the impurity molecules diffusing from the solution 2) migration of impurity molecules across the surface and 3) step/kink adsorption of impurity molecules migrating on the surface.

These processes are associated with changes of thermodynamic energy, whose relative values determine if and how adsorption occurs. The coverage of active sites by an impurity, θ_l, will be proportional to overall surface coverage, θ_s. It is represented by:

$$\theta_l = \beta \theta_s \qquad \text{(S5)}$$

The parameter β can take high values when the second and third steps are thermodynamically favorable. For highly mobile impurities β is greater than one, indicating that the impurities concentrate on the active sites (kinks). For favorable adsorption on the surface, β takes small positive values ($0<\beta<<1$).[1] Assuming that Burton, Cabrera and Frank diffusion mechanism[2,3] describes the crystal growth, the ratio between linear growth rate in impure, G, and pure, G_o, solutions is given[1]:

$$\frac{G}{G_o} = 1 - \theta_l \qquad \text{(S6)}$$

Replacing the equations S2 and S5 at S6 is obtained:

$$\frac{G}{G_o} = 1 - \beta_i \frac{k_i c_{imp}}{k_i c_{imp} + k\sigma + 1} \qquad \text{(S7)}$$

In case of low surface coverage, low k_i, high solute solubility, c_s (high k and low k_i) and low impurity concentration, the impurity is called barely adsorbed impurity. Impurities that affect the crystal growth rate are considered active impurities[1].

"Reprinted (adapted) with permission from {Martins, P. M.; Rocha, F. A.; Rein, P. The Influence of Impurities on the Crystal Growth Kinetics According to a Competitive Adsorption Model. *Crystal Growth & Design* **2006**, *6* (12), 2814–2821. https://doi.org/10.1021/cg060448x.}. Copyright {2023} American Chemical Society."

References

(1) Martins, P. M.; Rocha, F. A.; Rein, P. The Influence of Impurities on the Crystal Growth Kinetics According to a Competitive Adsorption Model. *Crystal Growth & Design* **2006**, *6* (12), 2814–2821. https://doi.org/10.1021/cg060448x.

(2) Burton, W. K.; Cabrera, N.; Frank, F. C.; Mott, N. F. The Growth of Crystals and the Equilibrium Structure of Their Surfaces. *Philosophical Transactions of the Royal Society of London. Series A, Mathematical and Physical Sciences* **1951**, *243* (866), 299–358. https://doi.org/10.1098/rsta.1951.0006.

(3) Uwaha, M. Introduction to the BCF Theory. *Progress in Crystal Growth and Characterization of Materials* **2016**, *62* (2), 58–68. https://doi.org/10.1016/j.pcrysgrow.2016.04.002.
